# In Vitro and In Vivo Characterization of Dibenzothiophene Derivatives [^125^I]Iodo-ASEM and [^18^F]ASEM as Radiotracers of Homo- and Heteromeric α7 Nicotinic Acetylcholine Receptors

**DOI:** 10.3390/molecules25061425

**Published:** 2020-03-20

**Authors:** Cornelius K. Donat, Henrik H. Hansen, Hanne D. Hansen, Ronnie C. Mease, Andrew G. Horti, Martin G. Pomper, Elina T. L’Estrade, Matthias M. Herth, Dan Peters, Gitte M. Knudsen, Jens D. Mikkelsen

**Affiliations:** 1 Neurobiology Research Unit, Copenhagen University Hospital, Rigshospitalet, DK-2100 Copenhagen, Denmark; hbh@gubra.dk (H.H.H.); Hanne.D.Hansen@nru.dk (H.D.H.); elina.nyberg@sund.ku.dk (E.T.L.); Gitte.Knudsen@nru.dk (G.M.K.); 2 Department of Brain Sciences, Imperial College London, London W12 0 LS, UK; 3 Russell H. Morgan Department of Radiology and Radiological Science, The Johns Hopkins University School of Medicine, Baltimore, MD 21287, USA; rmease1@jhmi.edu (R.C.M.); ahorti1@jhmi.edu (A.G.H.); mpomper@jhmi.edu (M.G.P.); 4 Department of Drug Design and Pharmacology, University of Copenhagen, Jagtvej 162, 2100 Copenhagen, Denmark; matthias.herth@nru.dk; 5 Department of Clinical Physiology, Nuclear Medicine & PET, Rigshospitalet, Blegdamsvej 9, 2100 Copenhagen, Denmark; 6 DanPET AB, 216 19 Malmö, Sweden; info@danpet.eu

**Keywords:** alpha 7, nicotinic acetylcholine receptors, PET, nAChR, autoradiography

## Abstract

The α7 nicotinic acetylcholine receptor (α7 nAChR) is involved in several cognitive and physiologic processes; its expression levels and patterns change in neurologic and psychiatric diseases, such as schizophrenia and Alzheimer’s disease, which makes it a relevant drug target. Development of selective radioligands is important for defining binding properties and occupancy of novel molecules targeting the receptor. We tested the in vitro binding properties of [^125^I]Iodo-ASEM [(3-(1,4-diazabycyclo[3.2.2]nonan-4-yl)-6-(^125^I-iododibenzo[b,d]thiopentene 5,5-dioxide)] in the mouse, rat and pig brain using autoradiography. The in vivo binding properties of [^18^F]ASEM were investigated using positron emission tomography (PET) in the pig brain. [^125^I]Iodo-ASEM showed specific and displaceable high affinity (~1 nM) binding in mouse, rat, and pig brain. Binding pattern overlapped with [^125^I]α-bungarotoxin, specific binding was absent in α7 nAChR gene-deficient mice and binding was blocked by a range of α7 nAChR orthosteric modulators in an affinity-dependent order in the pig brain. Interestingly, relative to the wild-type, binding in β2 nAChR gene-deficient mice was lower for [^125^I]Iodo-ASEM (58% ± 2.7%) than [^125^I]α-bungarotoxin (23% ± 0.2%), potentially indicating different binding properties to heteromeric α7β2 nAChR. [^18^F]ASEM PET in the pig showed high brain uptake and reversible tracer kinetics with a similar spatial distribution as previously reported for α7 nAChR. Blocking with SSR-180,711 resulted in a significant decrease in [^18^F]ASEM binding. Our findings indicate that [^125^I]Iodo-ASEM allows sensitive and selective imaging of α7 nAChR in vitro, with better signal-to-noise ratio than previous tracers. Preliminary data of [^18^F]ASEM in the pig brain demonstrated principal suitable kinetic properties for in vivo quantification of α7 nAChR, comparable to previously published data.

## 1. Introduction

The α7 nicotinic acetylcholine receptor (α7 nAChR) belongs to the superfamily of ligand-gated ion channels and is expressed across all mammalian species [1,2,3,4]. The receptor plays an important role in cognition [5], mood [6] and consistent with this, α7 nAChR are particularly abundant in hippocampus and prefrontal cortex [7,8]. Furthermore, α7 nAChR are implied in neuro-immune [9] and immune functions [10] under homeostatic conditions.

Changes in protein and mRNA levels of α7 nAChR have been reported in a number of neuropsychiatric and neurodegenerative diseases [1,11,12,13,14,15]. Notably, certain polymorphisms in the promoter region of the α7 nAChR gene (*CHRNA7*) [16] are probable risk factors for neuropsychiatric diseases, such as major depression [17] and schizophrenia [18] and are associated with developmental disorders and cognitive impairments [19]. Additionally, α7 nAChRs are expressed by several central and peripheral immune cells and activation via agonists and positive allosteric modulators showed neuroprotective and immunomodulatory efficacy in different preclinical disease models [20,21,22,23,24].

Changes of α7 nAChR in the healthy and diseased brain can only be detected in vivo by molecular imaging, such as positron emission tomography (PET) using specific radiotracers. A clinically usable radiotracer requires sufficient selectivity, specificity and suitable affinity, depending on the target [25]. Most of the previously described α7 nAChR PET tracers, among those [^18^F]NS14490, [^11^C]NS14492, [^11^C]CHIBA-1001 and [^11^C]A-582941 (Table 1), studied in mice, pigs and non-human primates exhibited some shortcomings, such as poor specific and/or high nonspecific binding or radiometabolites crossing the blood–brain barrier [26,27,28,29,30,31,32]. Furthermore, the specificity of novel α7 nAChR tracers has not always been tested in respective gene-deficient mice, e.g., using in vitro autoradiography.

While development of novel tracers from different lead structures is still ongoing [33,34,35,36,37], tilorone [38] provided a lead-structure for a number of derivatives subsequently developed into α7 nAChR PET tracers. From those, [^18^F]ASEM (JHU82132) [39] and the structurally related [^18^F]DBT-10 (JHU82108) [40] have been most widely investigated. Initial studies have shown that ASEM is a potent antagonist [39] with subnanomolar affinity and high selectivity [39,41], further substantiated by the radiolabelled compounds [^18^F]ASEM [26] and [^125^I]Iodo-ASEM [42] as tested in human and rat recombinant α7 nAChR. [^18^F]ASEM and [^125^I]Iodo-ASEM readily enter the mouse brain, are displaceable, and accumulate in regions with highest α7 nAChR density [26,39,42,43].

More recent studies using [^18^F]ASEM and [^18^F]DBT-10 further supported the suitability of the tracers, showing high and reversible brain uptake with a regional binding pattern consistent with the distribution of α7 nAChR receptors in the non-human primate brain [39,44,45]. Favourable brain pharmacokinetics, excellent test-retest reproducibility and regional uptake [^18^F]ASEM pattern consistent with post-mortem α7 nAChR distribution have been reported in human PET studies [43,46]. Several recent studies extended the available data in human subjects, showing good agreement with previous distribution volumes (V_T_) and test-retest values in nonhuman primates and healthy volunteers [44]. A study in ageing subjects showed a significant positive correlation between age and [^18^F]ASEM V_T_ in striatum and several cortical regions [47], however without any correlation between V_T_ and cognitive measures. A small sample of individuals with schizophrenia on stable antipsychotic medication showed lower [^18^F]ASEM V_T_ in cingulate cortex and hippocampus [46] and individuals with recent onset of psychosis were also reported to show lower [^18^F]ASEM V_T_ in hippocampus, after controlling for age [48]. Interestingly, patients with mild cognitive impairment showed higher [^18^F]ASEM V_T_ when adjusted for age as compared to the control group [49], consistent with post-mortem findings from patients and animal models. Additionally, [^18^F]ASEM has been employed in a rat 6-OHDA lesion model of Parkinson’s disease, showing an initial increase of [^18^F]ASEM SUVr in the ipsilateral striatum and substantia nigra between 3 and 7 days, which coincided with several histology markers of glia activation [50].

While this data shows the general applicability of [^18^F]ASEM, binding properties and interpretation of novel α7 nAChR tracers might be complicated by the fact that α7 subunits can form heteromeric receptors together with other subunits, specifically β2 [51]. These receptors can be heterologously expressed in oocytes and are found in the rodent and human basal forebrain and cortex [52,53]. While these heteromeric receptors display different pharmacological properties [52,54], it is not clear how this translates to radiotracer binding. In vitro binding studies of [^18^F]ASEM or [^125^I]ASEM in gene-deficient mice could answer the question, and would also reveal the suitability of [^125^I]ASEM for in vitro autoradiographic studies. The latter would offer a better comparability to in vivo PET data over the current gold-standard tracer [^125^I]α-bungarotoxin. We therefore investigated the potential of [^125^I]ASEM for in vitro studies of the α7 nAChR, by comparing binding of [^125^I]Iodo-ASEM in rat, mouse and pig brain sections. Furthermore, [^18^F]ASEM was characterized for in vivo brain uptake and target selectivity in a PET study conducted in the pig.

## 2. Results

### 2.1. In Vitro Autoradiography

**Figure 1:**
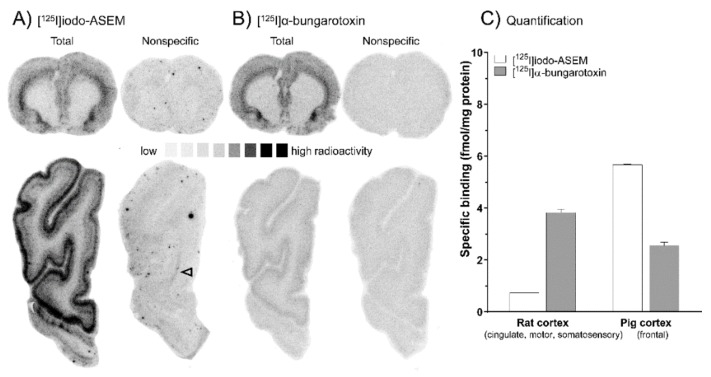
(**A**,**B**) Representative autoradiographs showing [^125^I]Iodo-ASEM and [^125^I]α-bungarotoxin total and non-specific binding (determined with 1 mMol/L (-)-nicotine for [^125^I]α-bungarotoxin and 10 µMol/L SSR-180,711 for [^125^I]Iodo-ASEM) in 12 µm sections of the rat (upper row) and pig brain (lower row). Arrowheads indicate residual white matter binding. (**C**) Comparative quantitative analysis of specific binding (± S.E.M.) of [^125^I]Iodo-ASEM and [^125^I]α-bungarotoxin from autoradiography in the rat (n = 1) and pig cortex (*n* = 2). All autoradiographic experiments and quantifications are carried out in 3-4 sections per animal.

**Figure 2:**
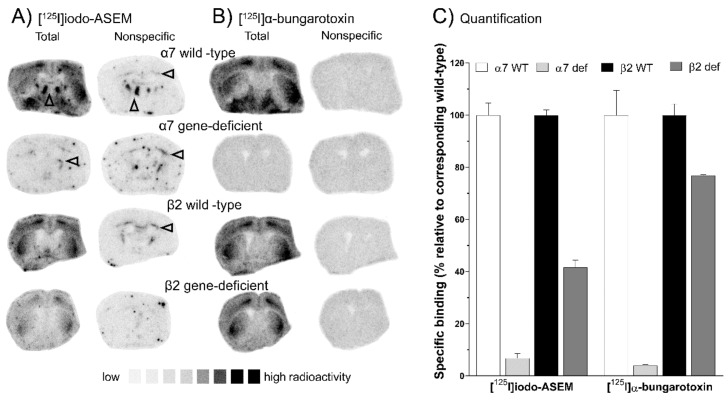
(**A**,**B**) Representative autoradiographs showing total [^125^I]Iodo-ASEM (**A**) and [^125^I]a-bungarotoxin (**B**) and non-specific (determined with 1 mMol/L (-)-nicotine for [^125^I]α-bungarotoxin and 10 µMol/L SSR-180,711 for [^125^I]Iodo-ASEM) binding in 12 µm brain sections of α7 and β2 nAChR wild-type vs. corresponding gene-deficient (def) mice (*n* = 1 each). Arrowheads indicate residual white matter binding. (**C**) Comparative quantitative analysis of specific binding (± S.E.M.) of [^125^I]Iodo-ASEM and [^125^I]a-bungarotoxin in α7 and β2 nAChR wild-type vs. corresponding gene-deficient mice (*n* = 1). All autoradiographic experiments and quantifications are carried out in 3-6 sections per animal.

Cerebral binding of [^125^I]Iodo-ASEM was investigated across several mammalian species, i.e., rat (Figure 1A, upper row), mouse (Figure 2A) and pig brain (Figure 1A, lower row) and compared to [^125^I]α-bungarotoxin (Figure 1B/2B). Total cortical [^125^I]Iodo-ASEM binding was highest in the pig, and lower in the rat and mouse (Figure 1C and Figure 2C), as compared to [^125^I]α-bungarotoxin. [^125^I]Iodo-ASEM binding was displaceable with (-)-nicotine (1 mMol/L, data not shown) and SSR-180,711 (10 µMol/L, Figure 1A and Figure 2A, nonspecific binding) in all species.

A non-specific binding component remained detectable under the described experimental conditions at low levels in white matter structures (arrowheads, Figure 1A and Figure 2A). The distribution pattern of [^125^I]Iodo-ASEM binding in the rat, mouse and pig brain was comparable to that of [^125^I]α-bungarotoxin (Figure 1B and Figure 2B). In the pig, [^125^I]Iodo-ASEM showed a laminar binding pattern in the frontal cortex, with highest density in cortical layers (1–3) (Figure 1A, lower row), while in the rat, binding in motor, cingulate and somatosensory cortex was more prominent in layers 5–6. However, this species difference was also observed for [^125^I]α-bungarotoxin (Figure 1B).

The specificity of [^125^I]Iodo-ASEM to α7 nAChR is further substantiated by tracer binding experiments in α7 nAChR gene-deficient mice. Specific [^125^I]Iodo-ASEM binding was lacking in α7 nAChR gene-deficient mice (Figure 2A), as indicated by the overall reduction in total binding by 93% ± 1.7%, compared to wild-type animals (Figure 2C). Similarly, [^125^I]α-bungarotoxin total binding (Figure 2B) was 96% ± 0.4% lower in α7 nAChR gene-deficient mice (Figure 2C). In wild-type mice, no difference in [^125^I]Iodo-ASEM and [^125^I]α-bungarotoxin binding was observed (Figure 2A,B). However, traces of nonspecific binding were again noted in white matter structures (arrowheads in Figure 2A).

[^125^I]Iodo-ASEM binding in β2 nAChR gene-deficient mice was different compared to [^125^I]α-bungarotoxin. An overall 58% ± 2.7% lower specific [^125^I]Iodo-ASEM binding was observed, as compared to corresponding wild-type controls (Figure 2C). In contrast, [^125^I]α-bungarotoxin binding was reduced by 23% ± 0.2%, being less affected by β2 nAChR gene-deficiency as compared to the reduction in [^125^I]Iodo-ASEM binding.

**Figure 3:**
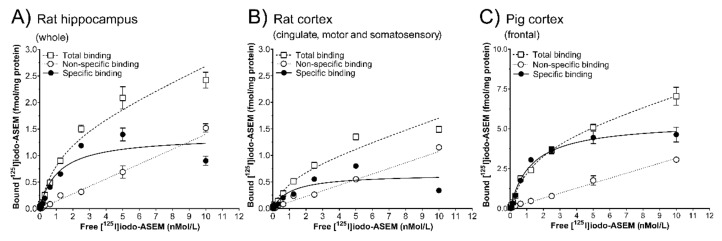
Saturation binding of [^125^I]Iodo-ASEM (0.02-10 nMol/L) to 12 µm sections from the rat hippocampus and cortex (**A**, **B**, *n* = 1) and pig frontal cortex (**C**, *n* = 1) brain. Non-specific binding was determined in the presence of 10 µMol/L SSR-180,711. Optical density of the autoradiograms was converted into ligand binding (fmol/mg protein ± S.E.M.) from a representative experiment. Data from saturation binding experiments were analysed by non-linear regression. Individual *K*_d_ and B_max_ values are indicated in Section 2.1. All autoradiographic experiments and quantifications are carried out in 2–4 sections per animal using 10 radioligand concentrations.

Saturation binding in rat and pig brain sections indicated that [^125^I]Iodo-ASEM binding was saturable. In the rat, non-linear regression analysis revealed an equilibrium dissociation constant (*K*_d_) of 1.14 nM (cortex, Figure 3B) and 1.17 nM (hippocampus, Figure 3A) with corresponding receptor density (B_max_) of 0.70 fmol/mg protein (cortex) and 1.44 fmol/mg protein (hippocampus), respectively (Figure 3A,B). In comparison, the pig cortex showed a *K*_d_ of 1.21 nM with a B_max_ of 5.47 fmol/mg protein (Figure 3C). The non-specific binding of [^125^I]Iodo-ASEM at concentrations near the *K*_d_ was low (rat hippocampus, 20%; rat cortex, 30%; pig cortex, 10%).

A range of selective α7 nAChR ligands (10 µMol/L each), including the α7 nAChR preferring antagonist methyllycaconitine (MLA), were used to test whether in vitro [^125^I]Iodo-ASEM binding (0.5 nMol/L) could be blocked in the pig cortex (Table 2). The partial agonists, NS14492, TC-5619, EVP-6124, A-582941, and SSR-180,711, showed almost complete (>90%) blocking of [^125^I]Iodo-ASEM binding in receptor dense areas of the cortex, e.g. layers 1–3. In contrast, GTS-21 (weak α7 nAChR agonist, ~70% reduction) and MLA (α7 nAChR preferring antagonist, ~80% reduction) exhibited less efficacious blockade of [^125^I]Iodo-ASEM binding in the pig cortex.

### 2.2. In Vivo PET Imaging in the Pig Using [^18^F]ASEM

**Figure 4:**
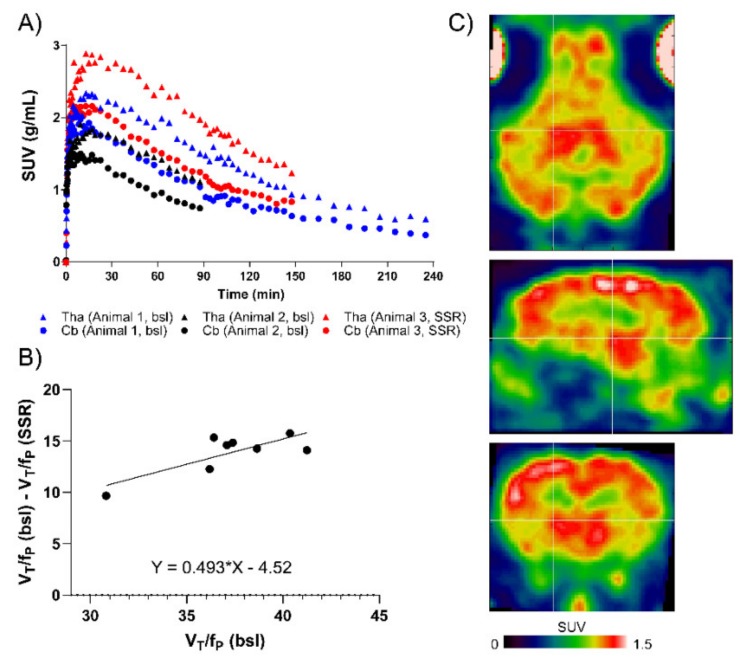
[^18^F]ASEM binding in the pig brain. (**A**) Time-activity curves of [^18^F]ASEM in three different animals: Animal 1, bsl, blue; Animal 2, bsl, black; Animal 3, SSR-180,711 pre-treated, red. The regions shown are: Thalamus (tha, triangles) and cerebellum (cb, circles). (**B**) Lassen plot with total distribution volumes (V_T_) corrected for free fraction in plasma (f_P_) using values from animal 1 and animal 3. Each point represents one region of interest (ROI), please refer to the method section for the complete list of ROIs. (**C**) Summed PET image (0–240 min) from animal 1 showing the distribution of [^18^F]ASEM in the pig brain. SUV: standard uptake value. Bsl: baseline. SSR: SSR-180,711 (1 mg/kg).

[^18^F]ASEM readily entered the pig brain and highest tracer accumulation was found in the thalamus followed by cortex, striatum and cerebellum (Figure 4A,C). [^18^F]ASEM uptake in the white matter was initially lower than in the grey matter regions, however the tracer kinetics were also slower, resulting in lower grey to white matter ratio at the end of the scans. The metabolism of [^18^F]ASEM in pigs was relatively slow, with 60% of the radioactivity at 120 min still being parent radioligand (data not shown). Kinetic modelling was performed to quantify the tracer uptake. Baseline V_T_ values varied between animals but after correcting for free fraction in plasma(f_P_), there was only a 5% difference in V_T_/f_P_ values between the two baseline animals (Table 3). This also suggest that V_T_/f_P_ values are unaffected by relatively large differences in injected mass (0.35 μg and 1.78 μg).

In a third animal, we evaluated the specificity of [^18^F]ASEM binding in vivo, by administering SSR-180,711 (1 mg/kg) prior to injection of [^18^F]ASEM. Compared to the baseline studies, we found an increase in [^18^F]ASEM uptake in all brain areas investigated (Figure 4A). Quantification of uptake and subsequent correction for f_P_ revealed that SSR-180,711 administration decreased the V_T_/f_P_ compared to baseline (Table 3). Occupancy was computed with the Lassen plot using V_T_/f_P_ values comparing baseline data from animal 1 and blocking data from animal 3 (0-150 min scan data). We found that the 1 mg/kg SSR-180,711 dose resulted in a 49% occupancy (Figure 4B).

From the Lassen plot, the volume of non-displaceable binding (V_ND_/f_P_) was found to be 9.2 mL/cm^3^. When comparing the V_ND_/f_P_ to the V_T/_f_P_ in the thalamus, we found that 78% of the signal observed in the thalamus is specific binding, leaving 22% as non-displaceable binding.

In one animal, [^18^F]ASEM acquisition time was 240 min, which allowed subsequent analysis of the time-stability of the parameters estimated with kinetic modelling. Again, the LGA model was used to determine V_T_ with different scan length and V_T_ values were found to decrease with more time included in the kinetic modelling. Using all data (0–240 min), V_T_ values were 5.4 mL/cm^3^ (thalamus), 5.0 mL/cm^3^ (frontal cortex) and 4.0 mL/cm^3^ (cerebellum).

The upper half of the table shows the baseline distribution volumes (V_T_) values with and without correction for free fraction in plasma (f_P_) in two different animals. Bottom part of the table describes V_T_ values with and without correction for (f_P_) at baseline (animal 1) and after pre-treatment with SSR-180,711 (animal 3)**.** See Table 4 (Material and Methods) for f_P_ values in the individual animals. Because animal 2 was only scanned for 90 min, the acquisition time of animal 1 was truncated to 90 min to allow for comparison.

## 3. Discussion

In this study, we investigated binding properties of radiolabelled ASEM in vitro ([^125^I]Iodo-ASEM] and in vivo ([^18^F]ASEM). Autoradiography was used to determine the applicability of [^125^I]Iodo-ASEM for in vitro assessment of α7 nAChR receptor distribution and occupancy in the mammalian brain. [^125^I]Iodo-ASEM showed high-affinity and specific binding to α7 nAChR in the rat, mouse and pig brain. Specific binding was absent in α7 gene-deficient mice, indicating high specificity and selectivity. Saturation binding experiments in rat and pig brain sections revealed low nanomolar *K*_d_ values (approximately 1 nM) in both species. B_max_ in the pig cortex was considerably higher as compared to the binding found in the mouse and rat brain cortex. Such species differences are well documented in the literature, e.g., for metabotropic glutamate 5 receptors and the 18 kDa translocator protein between monkey and humans [55,56]. As affinity and selectivity are major criteria for radiotracers, our data further substantiates the suitability of ASEM derivatives as favourable α7 nAChR tracers [57,58]

We found that the affinity of [^125^I]Iodo-ASEM in the pig brain as determined with autoradiography was in a similar range as [^3^H]NS14492 [59]. In contrast, higher affinities and receptor densities are reported for radioligand binding assay in brain homogenates for a number of different α7 nAChR ligands, such as [^3^H]NS14492 and [^3^H]A-585539 [60], including other dibenzothiophenes [26,38].

Across the brain and specifically regions with high α7 nAChR expression, such as hippocampus and superficial cortical layers, non-specific binding of [^125^I]Iodo-ASEM at concentrations near the *K*_d_ was low (10%–30% of total binding) and produced a robust specific signal. However, a consistent nonspecific binding component in white matter was observed in all investigated species, in particular the corpus callosum and subcortical tracts. This is supported by previously reported in vivo findings in human and non-human primate subjects [39,43], where tracer uptake was lowest in white matter structures, such as the corpus callosum. Furthermore, we also observed slower in vivo kinetics in white matter structures in the pig brain. This could be caused by lower perfusion or kinetics may be different when the tracer interacts with lipid membranes, compared to interaction with the receptor. As in vitro binding conditions are distinctly different due to absent metabolism and blood flow, it is possible that these effects may limit pronounced non-specific white matter binding in vivo or that the nonspecific binding exhibits much slower kinetics. Under the employed incubation conditions, [^125^I]α-bungarotoxin shows no white matter residual binding. However, under the same conditions, the overall non-specific binding in grey matter for [^125^I]α-bungarotoxin is approximately 45% in human (data not shown) and 55% in pig brain tissue, where it is much lower for [^125^I]Iodo-ASEM (~10%–30%).

[^125^I]Iodo-ASEM binding enables an important distinction between grey and white matter structures, e.g., the distinct cortical laminar binding pattern observable in the pig. While [^125^I]Iodo-ASEM binding was prominent in the deeper cortical layers in the mouse and rat, superficial cortical layers were intensely labelled in the pig. Using in vitro autoradiography, similar laminar cortical binding pattern in the pig brain has also recently been reported for a structurally different α7 nAChR radioligand, [^3^H]NS14492 [61]. Binding of both tracers was matching the pattern of [^125^I]α-bungarotoxin, the in vitro gold-standard radioligand for α7 nAChR. However, the spatial binding pattern in the rodent brain was only similar between [^125^I]Iodo-ASEM and [^125^I]α-bungarotoxin, but not for [^3^H]NS14492, suggesting different binding profiles of antagonists and agonists, or species differences in affinity.

[^125^I]Iodo-ASEM proved specific to the α7 nAChR, as evidenced by the lack of specific binding in α7 nAChR gene-deficient mice and a virtually complete block of cortical [^125^I]Iodo-ASEM binding by a wide range of structurally different α7 nAChR selective ligands and MLA, with the rank order being NS14492=TC-5619=EVP-6124=A-582941=SSR-180,711>MLA>GTS-21. While this corresponds well with the individual high affinities in the nanomolar range (NS14492, TC5619, EVP-6124, A-582941, SSR-180,711) [31,62,63,64,65], as compared to the lower affinity of a partial agonist (GTS-21) [66], it could also reflect the general differences between antagonists and agonist in terms of binding sites and kinetics.

When comparing to [^125^I]α-bungarotoxin, the specific binding of [^125^I]Iodo-ASEM was lower in the rat and mouse brain, but higher in the pig cortex. While species differences in receptor structure may account for the discrepancies, it should also be considered that different incubation protocols were used for the determination of optimal [^125^I]Iodo-ASEM and [^125^I]α-bungarotoxin binding. Hence, a relatively high detergent concentration was required in the experiments to obtain optimal total tissue binding of [^125^I]Iodo-ASEM, which may potentially affect binding of the radioligand in the mammalian species tested, e.g., through differences in lipid content and myelination. For example, [^125^I]Iodo-ASEM showed some degree of non-displaceable binding to white matter structures, which could be caused by the ligands’ lipophilicity and/or different kinetics in white matter structures.

Interestingly, radioligand binding in β2 nAChR gene-deficient mice was more strongly reduced for [^125^I]Iodo-ASEM than [^125^I]α-bungarotoxin. This observation suggests different binding properties and subtype selectivity to heteromeric α7β2 nAChR, compared to the homomeric receptors. In the CNS, heteromeric α7β2 nAChR are identified in the mouse forebrain and hippocampal neurons, rat basal forebrain cholinergic neurons, as well as in the human basal forebrain and cerebral cortex. Importantly, α7β2 nAChR display distinct functional properties as compared to homomeric α7 nAChR [51,67], owing to their slower whole cell decay kinetics and current amplitudes in both transfected cell systems and native rodent neurons [53,54,68,69,70]. Accordingly, co-expression of α7 and β2 nAChR subunits in *Xenopus* oocytes also results in lower maximal responses (evoked current amplitudes) of selective α7 nAChR agonists but does not shift pharmacology to a more β2-like profile [52,53,54,71]. These in vitro studies in transfected cell systems therefore suggest that α7 nAChR agonists bind to the α7-α7 subunit interface, and β2 subunits likely do not contribute to the ligand binding site on heteromeric α7β2 nAChR [67,71]. When using selective α7 nAChR antagonists, including MLA and α-bungarotoxin, to alter the response to some nicotinic agonists in either homomeric α7 and heteromeric α7β2 nAChR, results have been less consistent, as they show unaltered [54] or reduced potency [52] and efficacy [53] in comparison to homomeric α7 nAChR expressed in *Xenopus* oocytes. The functional significance of heteromeric α7 nAChR expression is not well understood, with recent work suggesting that this subtype combination might be more sensitive to inhibition by oligomeric amyloid β_1–42_ [68,69] and isoflurane [72], as compared to homomeric α7 nAChR. Our finding that binding of [^125^I]Iodo-ASEM, an antagonist, was markedly reduced in the forebrain of β2 gene-deficient mice therefore may suggest that [^125^I]Iodo-ASEM binds to heteromeric α7β2 nAChR in the brain, as opposed to [^125^I]α-bungarotoxin. Whether this is due to different affinity for homomeric α7 and heteromeric α7β2 nAChR requires further in vitro studies. Although speculative, this may offer a chance to probe the binding of amyloid β_1–42_ to heteromeric α7β2 nAChR in vivo using PET.

In vivo uptake of [^18^F]ASEM into the pig brain occurred rapidly within the first 10–20 min and a reversible but slower washout was found, as observed in human and non-human primate subjects [39,43]. The in vivo distribution of [^18^F]ASEM found here is very similar to that of [^11^C]NS14492 and importantly, also in accordance with the distribution of α7 nAChR in the pig brain [31,73]. Furthermore, our data with [^18^F]ASEM matches previous reports with the structurally similar analogue [^18^F]DBT-10 in piglets [40].

We found variations in brain uptake and f_P_ in the two baseline animals, and this result is consistent with the interpretation that lower f_P_ will lead to lower brain uptake [74]. Due to the limited number of animals in this study, this observation merits further investigations. Our finding is however supported by PET studies in non-human primates with [^18^F]ASEM and [^18^F]DBT-10, where V_T_/f_P_ was shown to be a more stable outcome measure than V_T_ [44,45]. This has also been shown for radiotracers binding to other neurotransmitter receptors [75].

V_T_ was found to increase slightly when the scan time was prolonged. This phenomenon was most pronounced in the thalamus and least pronounced in the white matter and is evident from the TACs (Figure 4A), where the ratio between e.g., thalamus and cerebellum was lower at 240 min than at 90 min. This finding is in contrast with the non-human primate and human data, where V_T_ was underestimated when reducing the PET data from 180 to 60 min [44]. Given that ASEM is an antagonist, it is unlikely that internalization of the receptor-ligand complex is an explanation for the decrease in V_T_. We cannot exclude that other receptor adaptations, such as (de)sensitization, could be responsible for this observation. Desensitization could occur if experiments were not conducted at tracer dose, i.e., but we did not attempt to identify the mass dose limit of unlabelled ASEM. Although the injected doses of ASEM varied in the two baseline animals, we only found a 5% difference in the calculated V_T_/f_P_, which suggest that the studies were conducted at tracer doses. The injected doses in this study (0.007–0.085 ug/kg) are higher than the doses used in the non-human primate evaluation of [^18^F]ASEM, where injected doses ranged from 0.009 to 0.056 μg/kg [44]. Further studies are needed to identify the mass dose limit of unlabelled ASEM.

While pre-treatment with 1 mg/kg SSR-180,711 resulted in an increased uptake of [^18^F]ASEM, kinetic modelling for quantification of tracer uptake showed that SSR-180,711 at this dose resulted in 49% occupancy. A similar phenomenon has also been reported in piglets when [^18^F]DBT-10 was blocked by the weak agonist NS6740, which was ascribed to a potential blood flow-driven effect of NS6740 leading to greater central uptake of [^18^F]DBT-10 [40]. The increased tracer uptake could also be a result of peripheral α7 nAChR binding sites having been blocked by SSR-180,771. The occupancy found by us is in line with previous work of Horti et al., reporting 39% and 81% occupancy for doses of 0.5 and 5 mg/kg SSR-180,711, respectively [39]. The occupancy computed in this study should be interpreted with care, as the baseline and blocking study is conducted in two different pigs. Due to the half-life of [^18^F]ASEM, it was not possible to conduct the study in the same animal on the same day. A further limitation to this in vivo study is the low number of PET scans and animals and thus we can only provide a descriptive presentation of the data, without statistical evaluations.

From our results, [^125^I]Iodo-ASEM therefore offers several advantages over [^125^I]α-bungarotoxin: 1) low nonspecific binding, 2) similar high affinity and selectivity and 3) in vivo applicability and direct comparison of PET data with autoradiographic data. The lower nonspecific binding of [^125^I]Iodo-ASEM is advantageous primarily in vitro, as it allows for a better signal-to-noise ratio over [^125^I]α-bungarotoxin at very similar affinities. While a low nonspecific binding would also be favourable under in vivo conditions (e.g., PET), having two nearly identical molecules as tracers offers interesting avenues, especially for preclinical studies. In vivo PET/SPECT imaging data can be acquired through [^18^F]ASEM, [^18^F]DBT10 or [^123^I]Iodo-ASEM and results can be validated or extended by using the advantages of in vitro autoradiography (e.g. resolution) with [^125^I]Iodo-ASEM.

In conclusion, [^125^I]-Iodo-ASEM is applicable for visualizing α7 nAChR binding in vitro, its binding is different between species, and may potentially bind to heteromeric α7β2 nAChR. In addition, [^18^F]ASEM is demonstrated to have suitable kinetic properties for in vivo quantification of α7 nAChR in the pig.

## 4. Materials and Methods

### 4.1. Compounds and Radioligands

[^125^I]Iodo-ASEM [(3-(1,4-diazabycyclo[3.2.2]nonan-4-yl)-6-(^125^I-iododibenzo[b,d]thiopentene 5,5-dioxide)] was labelled according to previously published procedures [42]. Mean molar activity was 59.94 ± 6.25 TBq/mmol. [^125^I]Tyr-54-mono-Iodo-α-bungarotoxin (81.4 TBq/mmol) was purchased from Perkin-Elmer (Skovlunde, Denmark). (-)-nicotine tartrate was purchased from Sigma-Aldrich (St. Louis, MO). Unlabelled ASEM and precursor for radiosynthesis was provided by DanPET (Malmoe, Sweden). The α7-selective ligands were purchased from Sigma-Aldrich (MLA) or provided by DanPET (NS11492) or NeuroSearch A/S (Copenhagen, Denmark) (SSR-180,711, TC-5619, EVP-6124, A-58294, and GTS-21.

### 4.2. Tissue Origin and Sectioning for In Vitro Autoradiography

All animal procedures were approved by the Danish Animal Experimentation Inspectorate (J. No. 2012-15-2034-00156) and treated in concordance with the European Communities Council Directive of 24th November 1986 (86/609ECC).

One female Sprague-Dawley rat (250 g, obtained from Charles River, Sulzfeld, Germany) was euthanized with an intraperitoneal overdose of pentobarbital, the brain was quickly removed and snap-frozen in −50 °C 2-methylbutane, then stored at −80 °C until further processing.

Mice deficient for the α7 subunits (The Jackson Laboratory) and β2 (Institut Pasteur, Paris, France) and their corresponding wild-type littermates were bred (C57BL/6J background) in an animal care facility at Virginia Commonwealth University. Brains from α7 and β2 gene-deficient mice and corresponding wild-type littermates were kindly provided by Dr. M. Imad Damaj (Dept. of Pharmacology and Toxicology, Virginia Commonwealth University, Richmond, VA, USA). 

One two-month old female Danish domestic pig (Landrace x Yorkshire x Duroc, 22 kg) was euthanized with an intravenous injection of pentobarbital, the brain was quickly excised, separated in two hemispheres and frozen on dry ice, before being stored at −80 °C.

All brain specimens were cut in 12 µm serial sections on a cryostat (Microm HM 500 OM, Walldorf, Germany), thaw-mounted onto Super Frost slides (Thermo Scientific, Hvidovre, Denmark), briefly air dried and stored at −80 °C until further processing. Protein concentration was determined from single or three sections with the Bio-Rad Protein Assay (Bio-Rad, Hercules, CA, USA) based on the method of Bradford [76].

### 4.3. In Vitro Autoradiography with [^125^I]Iodo-ASEM

Initial optimization of assay conditions was performed to maximize total binding while keeping non-specific binding low. Adjustments included buffer composition and pH, detergent concentration, wash and incubation time and temperature. An assay buffer with 50 mMol/L Tris-HCl pH 7.4, 21 °C (termed Tris-HCl buffer) provided best preserved tissue integrity and lowest non-specific binding, as compared to physiologic Tris, Tris-EDTA-EGTA or HEPES-KRH buffer (data not shown). For all further experiments, tissue from 1–2 animals was used, with experiments and quantifications carried out using 3–4 sections for pig and rat tissue and 3–6 sections for mouse tissue. Adjacent sections were used for autoradiography for all similar experiments (e.g. saturation binding). Sections were brought to room temperature and pre-incubated for 20 min in Tris-HCl buffer (pH 7.4, 21 °C), then incubated for 60 min in the same buffer (21 °C) containing 1.5% Triton X-100 (v/v) and 0.5-1.0 nMol/L [^125^I]Iodo-ASEM in a humidified chamber. Non-specific binding of [^125^I]Iodo-ASEM was assessed in the presence of 10 µMol/L SSR-180,711, added to the buffer. Blocking of [^125^I]Iodo-ASEM binding was investigated with a series of individual α7 nAChR selective compounds (10 µMol/L) added to the incubation buffer, i.e., methyllycaconitine (MLA) [77], SSR-180,711 [63], NS14492 [31], TC-5619 [64], EVP-6124 [65], A-582941 [62], and GTS-21 [66]. Following incubation, slides were rinsed in Tris-HCl buffer (pH 7.4, 21 °C), washed 2 × 5 min in Tris-HCl buffer (pH 7.4, 4 °C) and rinsed (5 sec) in ice-cold distilled water. Slides were gently dried under an air stream and exposed to 4% paraformaldehyde vapour overnight at 4 °C, followed by another drying step in a desiccator for 1 h. Adjacent sections were used for autoradiography. 

### 4.4. In Vitro Autoradiography with [^125^I]α-bungarotoxin

Slides were thawed at room temperature (21 °C) for 30 min, followed by 30 min of rehydration in 50 mMol/L Tris buffer with 0.1% BSA (w/v), pH 7.3 (binding buffer). For assessment of α-bungarotoxin binding, the binding buffer contained 0.5 mMol/L [^125^I]α-bungarotoxin and 4.5 nMol/L unlabelled α-bungarotoxin (Tocris, Denmark) yielding a total of 5 nMol/L α-bungarotoxin (incubation buffer). Total binding was determined using one set of slides incubated with the radioligand for 2 h at room temperature in a humidified chamber. Non-specific binding was determined in the presence of 1 mMol/L (-)-nicotine added to the incubation buffer. Afterwards, slides were briefly rinsed in binding buffer, followed by 2 × 30 min of washing in ice-cold binding buffer (4 °C). Finally, slides were briefly rinsed (5 sec) in ice-cold distilled water, dried under a gentle air stream and exposed to 4% paraformaldehyde vapour overnight at 4 °C. On the next day, the slides were dried for 1 h in a desiccator. 

### 4.5. Saturation Binding and Kinetic Analysis Using In Vitro Autoradiography

Saturation binding was carried out in rat and pig brain sections as described above (Section 4.3). Sections were incubated with ten serial dilutions of [^125^I]Iodo-ASEM ranging from 0.02 to 10 nMol/L, with concentrations measured by gamma-counting. Non-specific binding was determined in adjacent sections in the presence of 10 µMol/L SSR-180,711 for each radioligand concentration. Binding was terminated by washing the sections in ice-cold binding buffer. The equilibrium dissociation constant (*K*_d_) and maximum number of binding sites (B_max_) were determined by non-linear regression analysis of a one-site saturation binding model using GraphPad Prism 6.0 (GraphPad Software, Inc., San Diego, CA, USA).

### 4.6. Autoradiographic Image Acquisition and Analysis

BAS SR2040 phosphor imaging plates (Fujifilm, Toyko, Japan) were exposed to the samples along with [^125^I] standards (ARI 0133A; American Radiolabeled Chemicals, St. Louis, USA) for 24-72 hours. Imaging plates were scanned using a Phosphor Imager BAS-2500 (Fujifilm Europe GmbH, Düsseldorf, Germany). Images were converted to TIF-files using the manufacturer’s software and analysed in QuantityOne (BioRad, Waltham, MA, USA). Regions of interest (ROIs) were drawn over grey and white matter structures, depending on the investigated species. High intensity circular spots were occasionally observed in [^125^I]Iodo-ASEM autoradiographs and were excluded from the analysis. For α7 and β2 gene-deficient mice, only one ROI was drawn over the whole brain, again excluding spots and irregular white matter binding. In the rat brain, the ROIs were drawn over cortex and hippocampus. From the pig brain, only frontal cortex sections were cut, the ROIs therefore contained the frontal cortex and white matter tracts. The mean values of optical density per mm^2^ (averaged from the replicates) were converted to radioactive concentration using a linear regression derived from the [^125^I] radioactive standards. A global background of the imaging plate and individual non-specific binding were subtracted. Final values were expressed as fmol/mg protein, based on the protein measurements from individual sections.

### 4.7. Radiosynthesis of [^18^F]ASEM

The radiosynthesis of [^18^F]ASEM was performed as previously published [26]. No-carrier-added aqueous ^18^F-fluoride from the target was collected at a non-conditioned activated (10 mL ethanol, 20 mL water and dried with air) anion-exchange cartridge (QMA). A solution of 20 mg of 1,10-diaza-4,7,13,16,21,24-hexaoxabicyclo[8.8.8]hexacosane (Kryptofix-222) and 3.3 mg of K_2_CO_3_ dissolved in a 0.65 mL methanol-water mixture (97/3 v/v) was used to elute the ^18^F-fluoride off the cartridge. The elute was thereafter dried by evaporation at 90 °C under nitrogen and then further dried twice with 1 mL dry acetonitrile. To the dried Kryptofix^®^222/[^18^F]fluoride complex, 2.4 mg (0.006 mmol)/L of 3-(1,4-Diazabicyclo[3.2.2]nonan-4-yl)-6-nitrodibenzo[b,d]- thiophene 5,5-Dioxide dissolved in 0.8 mL DMSO was added. The reaction was performed at 160 °C for 15 min and afterwards the crude was quenched with 3.5 mL H_2_O. Reactants and by-products were separated from [^18^F]ASEM by semi-preparative HPLC [Luna column, Phenomenex Ltd. Aschaffenburg, Germany; 10 µm C18(2) 10×250 mm column, flow rate 6 mL/min, eluent: Ethanol/0.1% H_3_PO_4_ in water (25:75) with 6 mM ascorbic acid to prevent radiolysis]. The retention time for [^18^F]ASEM was 400-450 s and the product was collected into a vial containing 9 mL of PBS (phosphate-buffered saline). The product was visually inspected for clarity, absence of colour and visible particles. Chemical and radiochemical purities were assessed by analytical HPLC [Kinetex column, Phenomenex Ltd. Aschaffenburg, Germany; 2.6µ C18 4.60 × 50 mm, eluent: ACN/0.1% H_3_PO_4_ in water (25:75) RT: [^18^F]ASEM = 1.3 min; nitro precursor = 1 min; flow rate 1.5 mL/min]. Molar activity (A_m_) of the radiotracer was determined as follows: the area of the UV absorbance peak corresponding to the radiolabelled product was measured (integrated) on the HPLC chromatogram. This value was then converted into a molar mass by comparison with an average of integrated areas (triplet) of a known standard of the reference compound.

### 4.8. In Vivo Imaging in the Pig

Three female pigs (21, 22 and 23 kg) were used for in vivo PET imaging on a HRRT PET scanner (Siemens Healthcare, Erlangen, Germany). All animal procedures were approved by the Danish Council for Animal Ethics (journal no. 2012-15-2934-00156).

#### 4.8.1. Animal Procedures

Before scanning, anaesthesia was induced with i.m. injection of 0.13 mL/kg Zoletil veterinary mixture (Virbac, Kolding, Denmark; 10.87 mg/kg xylazine + 10.87 mg/kg ketamine + 1.74 mg/kg methadone + 1.74 mg/kg butorphanol + 10.87 mg/kg tiletamine + 10.87 mg/kg zolezepam). Hereafter, anaesthesia was maintained with constant propofol infusion (1.5 mg/kg/h intravenous (i.v.); B. Braun, Melsungen, Germany). An arterial i.v. catheter was employed for blood sampling from the right femoral artery and two venous i.v. catheters for injections were placed in the left and right mammary veins. During anaesthesia, animals were endotracheally intubated and ventilated. Vital parameters (heart rate, body temperature, blood pressure, oxygen saturation and end tidal CO_2_) were continuously monitored during the scan.

#### 4.8.2. PET Scanning

[^18^F]ASEM was given as intravenous i.v. bolus, with experimental details described in Table 4.

#### 4.8.3. Blood Sampling

During the first 30 min of the scans, radioactivity in the whole blood was continuously measured using an ABSS autosampler (Allogg Technology, Mariefred, Sweden) counting coincidences in a lead-shielded detector. Concurrently, arterial whole blood was sampled manually at times 2.5, 5, 10, 20, 30, 40, 50, 70, 89, 91, 120 and 150 min after injection of [^18^F]ASEM. Total radioactivity in plasma (500 µL) and whole blood (500 µL) was measured in a well counter (Cobra 5003; Packard Instruments, Meriden, CT, USA), which was cross-calibrated to the HRRT scanner and autosampler. All measurements of radioactivity were decay corrected to the time of radioligand injection.

#### 4.8.4. Metabolite Analysis

Radiolabelled parent compound and metabolites were determined by direct injection of plasma into a radio-HPLC system (Dionex Ultimate 3000; Thermo Fisher Scientific, Hvidovre, Denmark) configured for column switching. Manually drawn arterial whole blood samples were centrifuged (1500 g, 7 min, 4 °C), and plasma was filtered through a syringe filter (Whatman GD/X 13 mm or 25 mm, PVDF membrane, 0.45 µm pore size; Frisenette ApS, Knebel, Denmark) prior to the analysis by HPLC as previously described [78]. To increase sensitivity on gamma counts from samples with low levels of radioactivity, eluent from the HPLC was collected into fractions (10 mL) using a fraction collector (Foxy Jr FC144; Teledyne, Lincoln, NE, USA) and counted offline in a well counter (2480 Wizard^2^ Automatic Gamma Counter, Wallac Oy, Turku, Finland).

#### 4.8.5. Determination of Free Fraction

The free, non-protein bound fraction of [^18^F]ASEM in pig plasma, f_p_, was estimated using an equilibrium dialysis chamber method as previously described [79].

#### 4.8.6. Reconstruction and Pre-Processing of PET Data

150 -minute list-mode PET data were reconstructed in 58 dynamic frames (6 × 10, 6 × 20, 6 × 30, 6 × 60, 4 × 120, 14 × 300, 8 × 150, 8 × 300 s). One animal was scanned for 240 min using the mentioned framing protocol but adding 9 frames of 600 s). Images consisted of 207 planes of 256 × 256 voxels of 1.22 × 1.22 × 1.22 mm. A summed picture of all counts in the 150-min scan was reconstructed for each pig and used for co-registration to a standardized MRI-based atlas of the domestic pig brain, similar to that previously published [80]. The time activity curveds (TACs) were calculated for the following volumes of interest (VOIs): thalamus, striatum, hippocampus, cerebellum, white matter, frontal cortex, somatosensory cortex, occipital cortex, rest of the cortex. Radioactivity in all VOIs was calculated as the average of radioactive concentration (Bq/mL) in the left and right sides. Outcome measure in the TACs was calculated as radioactive concentration in VOI (in kBq/mL) normalized to the injected dose corrected for animal weight (in kBq/kg), yielding standardized uptake values (g/mL).

#### 4.8.7. Kinetic Modelling of PET Data

The PET imaging data were analysed with the Logan graphical analysis (LGA) model, using the metabolite corrected arterial plasma concentration to calculate the primary outcome measure: total distribution volume (V_T_). The secondary outcome measure was V_T_ values corrected for free fraction in plasma (V_T_/f_P_). The parent fraction curve for [^18^F]ASEM was fitted with a Watabe fit. Both curves were constrained to 1.0 at time = 0. Kinetic modeling was performed in PMOD version 3.0 (PMOD Technologies).

## Figures and Tables

**Table 1:** Common α7 nAChR ligands and their structure, previously evaluated as radiotracers.

Tracer	Structure
[^11^C]CHIBA-1001	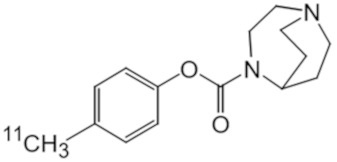
[^11^C]A-582941	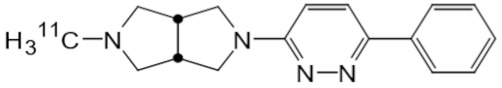
[^18^F]NS14490	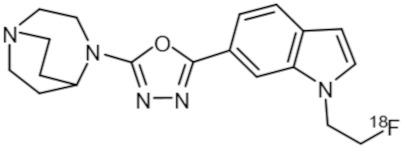
[^11^C]NS14492	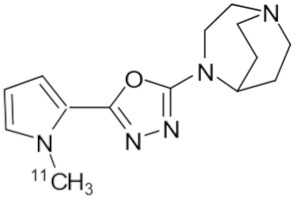
[^18^F]ASEM	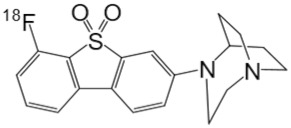
[^18^F]DBT-10	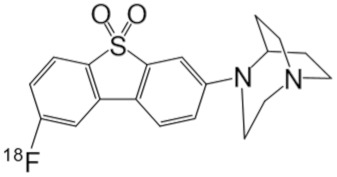
[^125^I]ASEM	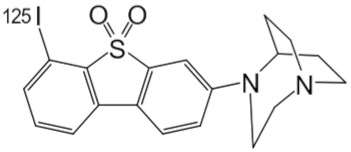

**Table 2:** Blocking of [^125^I]Iodo-ASEM binding in the pig cortex by a series of α7 nAChR ligands.

Ligand (10 µMol/L)	[^125^I]Iodo-ASEM Binding in the Pig Cortex, Layers 1–3 (%, mean ± S.E.M.)	[^125^I]Iodo-ASEM Binding in the Pig Cortex, Layers 4–6 (%, mean ± S.E.M.)
NS14492	4.04 ± 0.55	9.25 ± 0.83
TC-5619	7.88 ± 1.65	7.60 ± 0.30
EVP-6124	2.50 ± 0.20	5.50 ± 0.07
A-582941	3.22 ± 0.28	4.44 ± 0.93
SSR-180,711	2.92 ± 0.35	3.53 ± 0.50
GTS-21	30.92 ± 2.55	31.39 ± 2.15
MLA	20.46 ± 2.18	18.16 ± 2.41

Results are given in % remaining binding of total binding (mean ± S.E.M.).

**Table 3:** Kinetic modelling of [^18^F]ASEM with the Logan Graphical Analysis model in different pig brain regions.

Comparison of Baseline V_T_ Values.				
**Kinetic Modelling**	**Animal 1**		**Animal 2**	
**0–90 min**	**V_T_**	**V_T_/f_P_**	**V_T_**	**V_T_/f_P_**
Frontal cortex	7.87	43.70	3.75	41.66
Somatosensory cortex	8.33	46.27	4.15	46.14
Occipital cortex	8.03	44.63	3.77	41.86
Remaining cortex	7.63	42.37	3.78	41.94
Thalamus	8.83	49.06	4.12	45.73
Striatum	7.41	41.17	3.77	41.94
Hippocampus	7.53	41.83	3.59	39.93
Cerebellum	6.66	36.99	3.16	35.07
**Comparison of V_T_ Values at Baseline and After Pre-treatment with SSR-180,711**				
**Kinetic Modelling**	**Animal 1**		**Animal 3**	
**0–150 min**	**V_T_**	**V_T_/f_P_**	**V_T_**	**V_T_/f_P_**
Frontal cortex	6.73	37.38	3.61	22.57
Somatosensory cortex	7.26	40.35	3.94	24.60
Occipital cortex	6.96	38.65	3.90	24.40
Remaining cortex	6.68	37.08	3.60	22.50
Thalamus	7.42	41.24	4.34	27.15
Striatum	6.51	36.19	3.83	23.92
Hippocampus	6.55	36.41	3.37	21.09
Cerebellum	5.55	30.82	3.39	21.17

**Table 4:** Experimental details of [^18^F]ASEM PET scans in pigs.

Details	Animal 1	Animal 2	Animal 3
Type of experiment	Baseline	Baseline	SSR-180,711; 1 mg/kg
Scan length	240 min	90 min	150 min
Molar activity	20 GBq/µmol	345 GBq/µmol	388 GBq/µmol
Injected activity	99 MBq	335 MBq	189 MBq
Injected mass	1.78 μg	0.35 μg	0.18 μg
Free plasma fraction	18%	16%	9%
